# Expansion of Gutta-Percha

**Published:** 1903-08

**Authors:** Frank T. Taylor

**Affiliations:** Boston, Mass.


### EXPANSION OF GUTTA-PERCHA.

BY DR. FRANK T. TAYLOR, BOSTON, MASS.

The value of the expansive action of gutta-percha is often overlooked. Most of us have seen teeth which contained gutta-percha under metal fillings, and which had a weak side split off by the expansion of the capping.

Advantage can be taken of this expansive quality of gutta-percha by using it to separate teeth.

In many cases more space is needed than is easily secured by wedges in the ordinary way. In such cases pack the pink base-plate gutta-percha into the cavity until it completely fills both the cavity and the space between the teeth, pressing firmly against the adjacent tooth, or filling both cavities, if there are two.

This method is especially useful in cases where bicuspids and molars have moved together and space is required for properly contoured fillings.

The gutta-percha by its slower expansion, secures the necessary space in a few months without soreness of the teeth or annoyance to the patient.

The necessity of keeping the gutta-percha from crowding up into the gum and of protecting the pulp from the pressure of the expansion where the dentine covering is thin will be obvious.

